# Fermented Brown Rice Flour as Functional Food Ingredient

**DOI:** 10.3390/foods3010149

**Published:** 2014-02-12

**Authors:** Muna Ilowefah, Chiemela Chinma, Jamilah Bakar, Hasanah M. Ghazali, Kharidah Muhammad, Mohammad Makeri

**Affiliations:** UPM-BERNAS Research Laboratory, Faculty of Food Science and Technology, Universiti Putra Malaysia, 43400 UPM, Serdang, Selangor, Malaysia; E-Mails: mona.milad2005@gmail.com (M.I.); Chinmachiemela@yahoo.com (C.C.); jamilah@putra.upm.edu.my (J.B.); hasanah@putra.upm.edu.my (H.M.G.); makeri50@yahoo.com (M.M.)

**Keywords:** fermentation, brown rice flour, pasting properties, rheological properties, bread volume, texture properties

## Abstract

As fermentation could reduce the negative effects of bran on final cereal products, the utilization of whole-cereal flour is recommended, such as brown rice flour as a functional food ingredient. Therefore, this study aimed to investigate the effect of fermented brown rice flour on white rice flour, white rice batter and its steamed bread qualities. Brown rice batter was fermented using commercial baker’s yeast (Eagle brand) according to the optimum conditions for moderate acidity (pH 5.5) to obtain fermented brown rice flour (FBRF). The FBRF was added to white rice flour at 0%, 10%, 20%, 30%, 40% and 50% levels to prepare steamed rice bread. Based on the sensory evaluation test, steamed rice bread containing 40% FBRF had the highest overall acceptability score. Thus, pasting properties of the composite rice flour, rheological properties of its batter, volume and texture properties of its steamed bread were determined. The results showed that peak viscosity of the rice flour containing 40% FBRF was significantly increased, whereas its breakdown, final viscosity and setback significantly decreased. Viscous, elastic and complex moduli of the batter having 40% FBRF were also significantly reduced. However, volume, specific volume, chewiness, resilience and cohesiveness of its steamed bread were significantly increased, while hardness and springiness significantly reduced in comparison to the control. These results established the effectiveness of yeast fermentation in reducing the detrimental effects of bran on the sensory properties of steamed white rice bread and encourage the usage of brown rice flour to enhance the quality of rice products.

## 1. Introduction

The past two decades have seen a rapid increase in consumer demand for healthy foods, which has prompted recent research to find methods for production of healthy and functional foods. The usage of whole grain cereal instead of milled cereals is one such trend for production of healthy and functional foods, as consumption of whole grain foods has shown a reduction in the risk of several diseases, such as cardiovascular diseases, obesity, diabetes and some types of cancers [1,2]. One of the most significant components in the whole-cereal grains that play a significant part in its health properties is dietary fiber and phenolics, which are mainly concentrated in the outer layers of the cereal grain [1]. Production of whole grain foods is a complicated task for the food industry due to the mechanical negative effects of the bran on protein network formation and consequently on the sensory properties of the end product [3]. Accordingly, the most common forms of cereals composition are milled products, such as white wheat flour or white rice.

Rice is a unique crop due to its colorless, soft taste, low sodium levels, easy digestible carbohydrates and hypoallergenic properties. Therefore, its flour is an attractive food material to be used for making gluten free foods [4]. Steamed bread is a traditional product made from white rice and is known as Apam in Malaysia. It is very popular in this part of the world and consumed at breakfast. It is formulated with white rice flour, sugar, salt, water and yeast. This kind of product is an example of gluten free foods. 

Gluten free materials such as white rice flour do not have the required characteristics for production of leavened foods, since their proteins have no ability to develop a viscoelastic network such as gluten. In addition, lack of nutritional value has existed as a health problem of gluten free products specifically produced from white rice flour. Consequently, in recent years, there has been an increasing interest in using different food materials such as, gums, hydrocolloids and starches to enable developing a similar gluten network [5]. Moreover, whole grain flour such as millet, brown rice and sorghum were used to enrich gluten free foods [6,7]. Accordingly, using whole grain cereals flour could be promising for the development of healthy and acceptable gluten free foods.

Modification of whole-cereal flour prior to its usage by using simple food processing, such as fermentation, could eliminate the negative effects of the bran. Pre-fermentation of the whole-flour might increase fiber solubility due to enzyme reactions on the cell wall structure. The addition of pre-fermented wheat bran to wheat dough caused an increase in the bread volume of high fiber wheat bread [3]. The addition of yeast-fermented peeled bran to wheat bread increased its volume by 10%–15% and softened the crumb structure by 25%–35% compared to its unfermented bran [8]. Also, the addition of pre-fermented flour positively affected the texture, shelf life, aroma and nutritional value of gluten free foods, most probably because of metabolic activities of the microbes [9]. The positive effects associated with fermentation are the partial degradation of fiber and softening of bran particles [3]. The acidification rate of the dough is also an important property to achieve appropriate crumb structure and higher bread volume, since it affects the enzyme activities [10]. 

Recently, fermentation became a trend for production of healthy foods from whole grain cereals. Industrial application of the biotechnology of fermentation for the production of gluten free baked products is a promising innovation in the health foods industry. Thus, the objectives of the current study is to investigate the effect of fermented brown rice flour on white rice flour, white rice batter and its steamed bread (Apam) characteristics, which could encourage the usage of whole rice grains.

## 2. Materials and Methods

### 2.1. Materials

Brown rice grains (MR219) and commercial baker’s yeast (Eagle CY 1266, China) were bought from a local supermarket in Selangor, Malaysia. Brown rice grains were ground using a Cyclotech™ (1093) grinder (FOSS, Sweden) and a 500 µm sieve to obtain brown rice flour (BRF), which was used for the fermentation process. Brown rice grains were milled using a rice miller (Satake TM 05C, Australia) to obtain white rice grains that were ground to flour in a Cyclotech™ (1093) grinder (FOSS, Sweden) and sieved to produce white rice flour (WRF). Flour samples were packaged in polyethylene bags and stored at 4 °C. 

### 2.2. Fermentation Process

Fermentation conditions (time, temperature and yeast concentration) were optimized to achieve moderate acidity (pH 5.5) of fermented brown rice batter using Minitab 14 software (data not shown). Fermented brown rice batter at the optimum conditions was dried in an air oven drier at 50 °C for 3 h, ground to flour sieved in a 500 µm sieve and verified as fermented brown rice flour (FBRF).

### 2.3. Steamed Bread Making Process

**Figure 1:**
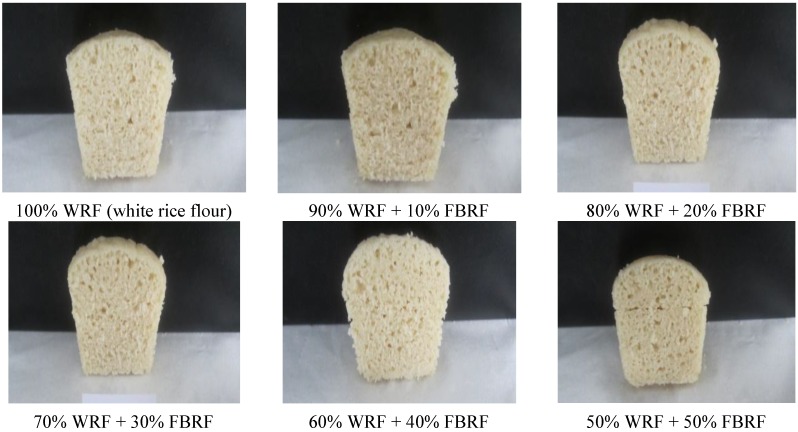
Digital images of steamed white rice bread (SWRB) and steamed white rice breads substituted with fermented brown rice flour (FBRF).

The formula of 100 g of WRF which contains 2% sugar, 2% salt, 3% baker’s yeast and 93% volume water based on the flour weight was used to prepare steamed white rice bread (SWRB). The SWRB formula of 60% white rice flour substituted with 40% FBRF achieved the highest bread volume and the overall acceptability of the tested sensory properties among the other ratios that included 0%, 10%, 20%, 30%, and 50% FBRF (data not shown) (Figure 1). To prepare rice bread; instant yeast was dissolved in a solution of water and sugar and conditioned at 32 °C for 10 min. Dry ingredients, such as WRF, salt and FBRF were mixed together, and then all the ingredients were mixed manually in a beaker for 3 min. White rice batter without FBRF was considered as the control. Finally, rice batters were positioned in cups and fermented in a fermenting box at 32 °C for 30 min. After fermentation, the samples were steamed for 15 min, cooled at room temperature (25 °C) for 1 h before measurements. Bread samples were made in five replicates.

### 2.4. Determination of Flour Pasting Properties

Pasting properties of WRF and WRF substituted with 40% FBRF was determined using a rapid viscos analyser (Newport Scientific Pty Ltd., New South Wales NSW, Australia) following the method of AACC 61-02 [11]. Peak viscosity, breakdown, final viscosity and setback of the samples were recorded by the Thermocline software.

### 2.5. Determination of Dynamic Rheological Properties of White Rice Batter

The dynamic rheological test was performed using a HAAKE Rheowin 600 rheometer, (Thermo, Germany) at 32 °C using parallel plate geometry (35 mm diameter and 1 mm gap). Batter samples were prepared as mentioned earlier, but without adding the yeast to avoid interference of air cells formation. After placing the batter samples between plates; batter samples were allowed to rest for 2 min to relax the residual stress. The linear viscoelastic part was measured by performing stress sweep experiment at 1 Hz frequency prior to determination. Based on the linear viscoelastic region, frequency sweep test was carried out at 1% strain between 0.1 and 10 Hz. Storage modulus (Gʹ), loss modulus (Gʺ), complex modulus (G*) and tan delta (δ) values were obtained from the Rheowin software (3.3). All the rheological tests were done in five replicates and their average values were stated in the results.

### 2.6. Measurement of Steamed Bread Volume

The volume of steamed breads was measured after 1 h of steaming according to the seed displacement method described by Hallén, *et al*. [12] using sago pearl. The loaf was inserted in a container with known volume (V_1_) and filled with sago pearl. After that, the volume of sago pearl used was recorded (V_2_). Bread volume (V) was calculated based on the following formula:

V (mL) = V_1_ − V_2_

Specific volume was determined by dividing the volume of the bread by its weight (cm^3^/g).

### 2.7. Texture Profile Analysis

The properties of the crumb texture of the breads were measured by using a Texture Analyzer (TA-XT2, UK) equipped with P/75 mm plate, 30 N load cell and trigger force of 5 g. After cooling at room temperature for 1 h, bread samples were prepared with approximately 2.5 × 2.5 × 2.5 cm dimensions taken from the crumb and compressed to 40% of its thickness [13], at a post and pre-speed of 2 mm/s and the interval time between first and second compression was 5 s. The texture properties, which include hardness, cohesiveness, chewiness, springiness and resilience were recorded using the Exponent software (32) (Stable Micro System, UK). All the samples were analyzed in five replicates.

### 2.8. Statistical Analyses

One way analysis of variance (ANOVA) and Tukey’s multiple range tests with a confidence interval of 95% were used to report the significant differences between the obtained results.

## 3. Results and Discussion

### 3.1. Pasting Properties of White Rice Flour (WRF) and White Rice Flour Supplemented with 40% FBRF

**Figure 2:**
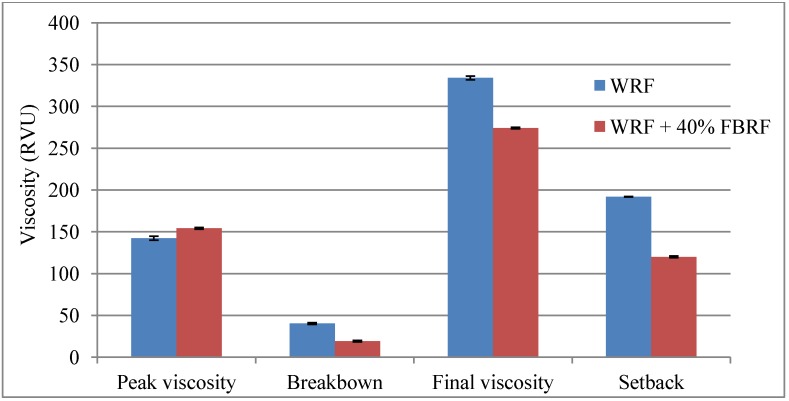
Pasting properties of white rice flour (WRF) and white rice flour with 40% FBRF.

Figure 2 shows the pasting properties of WRF and WRF with 40% FBRF. It can be observed that with addition of FBRF the peak viscosity significantly increased from 142.21 to 154.13 RVU, however, breakdown, final viscosity and setback were significantly reduced. The breakdown is associated with the ability of starch granules to be more resistant to being broken during heating and shearing. The results of this study indicated that the breakdown value was significantly reduced with the addition of FBRF. This result may be explained by the fact that, with an increase in protein content, some rice proteins could protect starch granules from being broken and increase pasting viscosity [14], as FBRF had a higher protein content after fermentation (data not shown). Also, the increase of peak viscosity and the decrease in breakdown values might indicate higher resistance to deformation and higher stability of the paste during baking. Moreover, the findings of the current study are consistent with those of Wang, *et al*. [15] who reported that final viscosity of rice paste decreased at low pH (4.10) and, in this study, the acidity of the rice batter with FBRF was higher than that of the control (data not shown). Another important finding was that the retrogradation phenomena could be significantly reduced in the final product, where setback—which is an indication of retrogradation phenomena—declined after addition of FBRF.

### 3.2. Viscoelastic Properties of White Rice Batter (WRB) and White Rice Batter Supplemented with 40% FBRF

**Figure 3:**
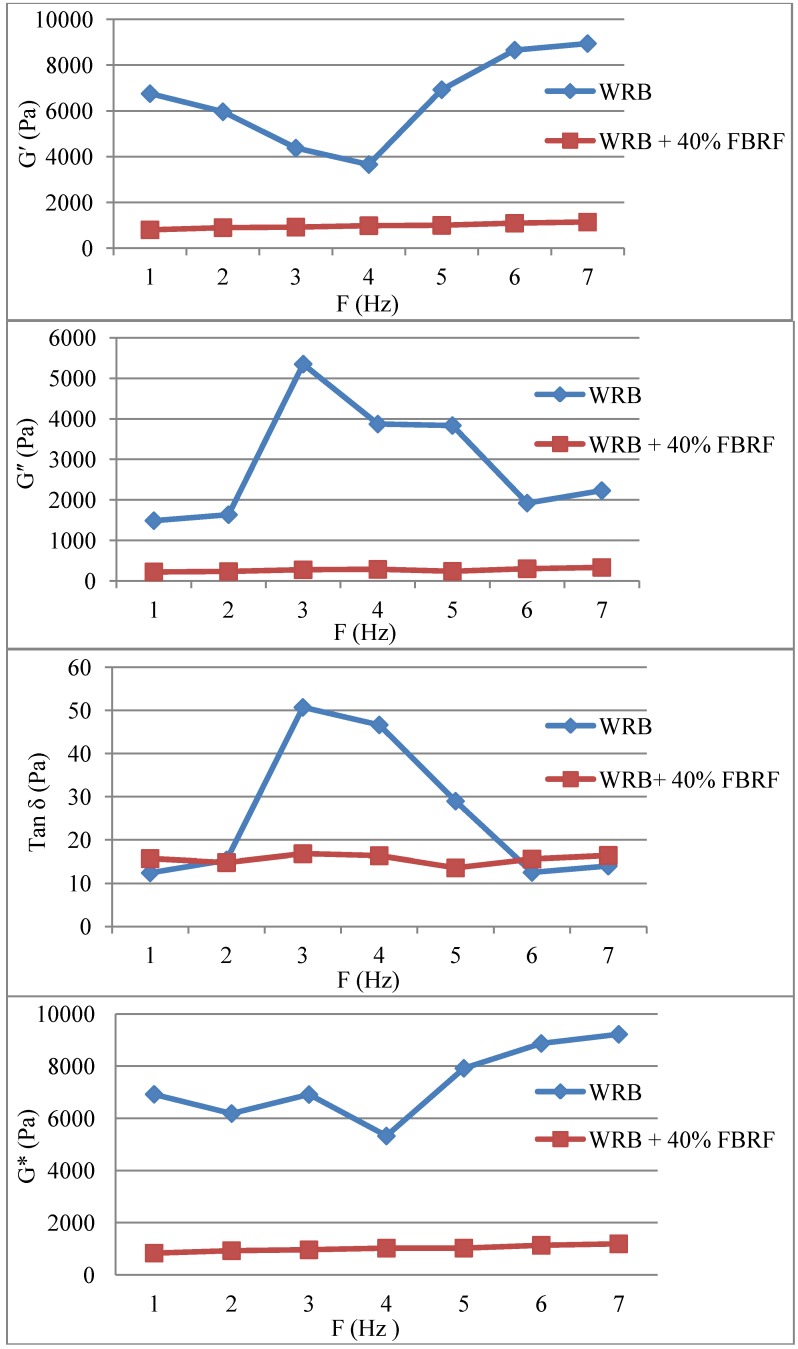
Viscoelastic properties of white rice batter and white rice batter with 40% FBRF.

The viscoelastic properties of the WRB and WRB containing 40% FBRF were studied using a dynamic oscillatory test. The mechanical spectra of the WRB sample indicated that both elastic (G′) and viscos module (G′′) values were higher than that of WRB with FBRF at all the tested frequency ranges (Figure 3). In addition, G′ and G′′ of WRB containing FBRF, independent of the frequency, compared to the reference sample. This suggested that the structure of WRB containing FBRF became softer and stronger than the control. It is reported that addition of acid to wheat dough reduced its extensibility and increased its resistance to extension [16]. It seems possible that these results are due to the rise in the positively charged proteins during acid treatment, where the pH of the batter containing FBRF was lower than that of the control. Nevertheless, acidity is not the only cause, since Rieder, *et al.* [16] reported that wheat dough containing fermented oat bran did not possess low pH, but exhibited higher resistance to extension and low extensibility. That means other factors associated with pre-fermented flour addition affected the dough rheology and thus the bread quality. The observed change in the viscoelastic properties could be attributed to the softening effect on the insoluble fiber due to the addition of FBRF. Rieder, *et al*. [16] also indicated that the addition of fermented barley flour to composite wheat bread degraded the fiber structure as indicated by a reduction in β-glucan molecular weight. In the current study, complex module decreased with addition of FBRF. However, the phase angle was not changed at low and high frequency, but decreased at 2.5 to 5.5 Hz (Figure 3). Another study reported that with addition of acetic and lactic acids to wheat dough, the results showed an obvious reduction in the complex modulus and the phase angle values [17].

### 3.3. Steamed Bread Volume

**Figure 4:**
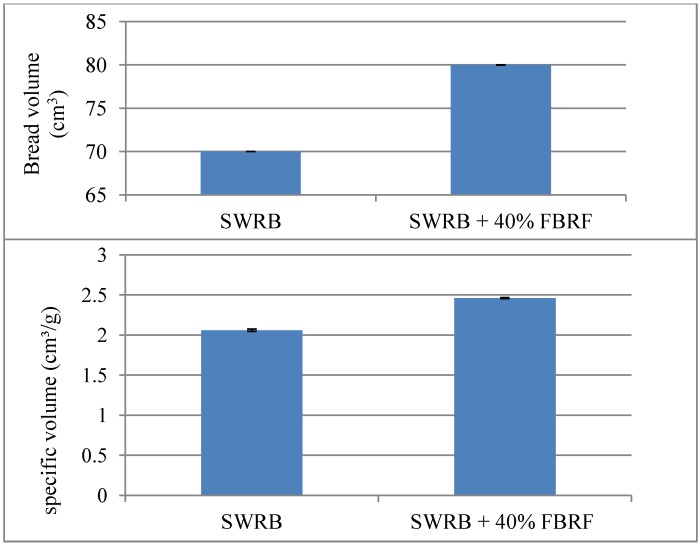
Volume and specific volume of steamed white rice bread (SWRB) and steamed white rice bread with 40% FBRF.

Figure 4 shows that SWRB containing 40% FBRF gained higher volume (80 cm^3^) and specific volume (2.46 cm^3^/g) in comparison to the reference sample (70 cm^3^ and 2.06 cm^3^/g, respectively). This finding supports previous research, where the addition of fermented barley flour to whole barley dough improved the overall quality of its bread. Its bread volume was significantly higher than that of the control [16]. Dough acidification is an important issue, since it affects bread volume, crumb softness and retrogradation phenomena. The reduction in the pH during dough fermentation activates some enzymes such as α-amylase and protease. During addition of pre-fermented flour to wheat dough the acidity performs on the gluten network, which could improve extensibility and softness of the dough that helped to retain higher amount of CO_2_ produced during fermentation, consequently increase the loaf volume [10]. However, high rate of acidity might increase hydrolyzation of the protein network; resulted in less elastic and softer dough that lead in a reduction in the bread volume, and elevate staling rate and bread firmness, as indicated by the addition of sourdough with high acidity to wheat dough [18]. The moderate decrease in the pH (5–6) of the dough because of microbial fermentation positively influences its structure specifically in high fiber breads, where addition of cereal fiber causes detrimental effects on the dough and bread structure. Also, the increase in bread volume of SWRB containing FBRF could be linked with the reduction in the loss and storage moduli of its batter and the reduction in breakdown of starch granules, which make the structure of the batter softer, stronger and more stable during steaming. 

### 3.4. Steamed Bread Texture

**Figure 5:**
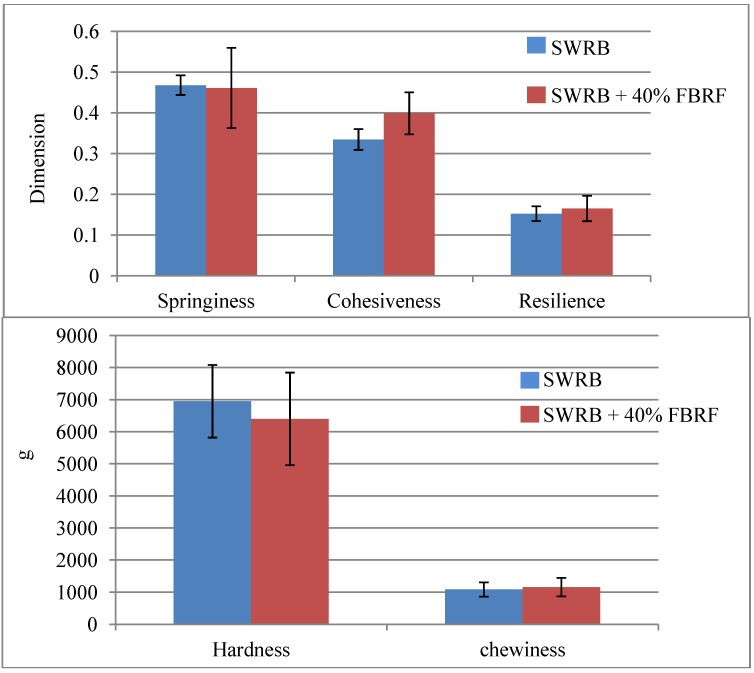
Texture properties of steamed white rice bread (SWRB) and steamed white rice bread with 40% FBRF.

Figure 5 shows the texture properties, which include hardness, springiness, cohesiveness, chewiness and resilience of the SWRB and SWRB supplemented by 40% FBRF. Hardness is the peak force during the first compression, and springiness or elasticity is defined as the height which the food recovered between the end of the first bit and beginning of the second bit. On the other hand, cohesiveness is the ratio of the positive force area during the second compression cycle to that during the first compression and adhesiveness is the negative force area for the first test and it is considered necessary to move the plunger away from the sample. Chewiness is assessed from the results of multiple analyses of gumminess and springiness [19]. The results of the current study indicated that the addition of FBRF softened the bread by recording less hardness value (6398.61 g) in comparison to the control (6948.13 g). The SWRB containing FBRF had higher values of chewiness, cohesiveness and resilience but a lower springiness value that indicated FBRF significantly improved the texture properties of SWRB. Rizzello, *et al*. [20] reported that pre-fermented fine and coarse bran fractions improved texture properties of the leavened baked product and the coarse fraction was therefore more effective. Fermented wheat germ is also observed to increase the bread volume of wheat bread and reduce its hardness rate [21]. Also, freeze-dried pre-fermented wheat germ had a positive influence on the texture and sensory characteristics of white wheat bread. The values of hardness and resilience of breads with fermented wheat germ were lower than those of the control, which means bread containing fermented germ was softer than its counterparts [21]. According to Salmenkallio-Marttila, *et al.* [3] addition of pre-fermented wheat bran to wheat bread supplemented with bran improved the crumb texture properties; specifically the elasticity. Also, it improved the bread volume and its shelf life. It is reported that adding pre-fermented bran with yeast and lactic acid bacteria improved the CO_2_ retention during dough proofing, and as a result increased the bread volume and the crumb softness [3,22].

## 4. Conclusions

Addition of fermented brown rice flour to steamed white rice bread significantly improved rheological and textural properties as well as volume of the bread as indicated in this study. Currently, investigation of the effect of fermented brown rice flour on the nutritional value of steamed white rice bread is under way.
